# Class III Orthodontic Camouflage: Is the “Ideal” Treatment Always the Best Option? A Documented Case Report

**DOI:** 10.1155/2022/9200469

**Published:** 2022-07-12

**Authors:** Lorenzo Rustico, Vincenzo Ronsivalle, Flavia Iaculli, Gianrico Spagnuolo, Gaetano Isola, Antonino Lo Giudice

**Affiliations:** ^1^Department of Medical-Surgical Specialties, School of Dentistry, University of Catania, Policlinico Universitario “G. Rodolico”, Via Santa Sofia 78, 95123 Catania, Italy; ^2^Department of Neurosciences, Reproductive and Odontostomatological Sciences, University of Naples “Federico II”, Via Pansini 5, 80131 Naples, Italy

## Abstract

Angle's Class III is one of the most complex malocclusions to treat. In nongrowing skeletal class III malocclusions, the choice between orthognathic surgery and camouflage treatment remains a challenge to the orthodontist. In class III borderline cases, clinicians are called to find the best compromise between functional and aesthetics outcomes, with the latter which often turns in avoiding worsening of profile characteristics, which makes the treatment of these patients quite challenging. This case report describes a borderline nongrowing patient with skeletal class III malocclusion, upper incisor proclination and spacing, lower crowding, and arch width discrepancy, which has already undergone previous orthodontic treatment. The orthodontic treatment involved the mandibular first premolar extraction, resulting in class I canine relation with good overjet and overbite as well as good arch coordination. The orthodontic camouflage improved the dental relationship with normalization of upper incisor inclination without a relevant retroclination of lower incisors; the skeletal facial pattern of the patient experienced a slight improvement. The tendency to skeletal class III has remained nearly unaffected. Treatment outcomes were stable after 1-year posttreatment follow-up.

## 1. Introduction

Angle's Class III is one of the most complex malocclusions to treat, and it is usually characterized by combined skeletal and dentoalveolar features [1–3].

The combination of a normal maxilla with a prognathic mandible has been identified by Jacobson et al. as the most frequent class III malocclusion pattern [4].

Angle's Class III correction can be accomplished by growth modification [5, 6], orthodontic camouflage [7], or orthodontic surgery [8].

Adult nongrowing Class III patients are even more challenging to treat due to the limited availability of treatment options. The decision between orthodontic camouflage and orthognathic surgery remains a trial for the orthodontist [9].

The best treatment option for nongrowing skeletal class III patients is represented by orthognathic surgery; however, this option is often refused by the patients due to finances or the invasiveness related to the procedure. Class III patients who refuse orthognathic surgery can be treated, with an orthodontic camouflage, extractions, utilising multibrackets and class III elastics, multiloop edgewise archwire treatment, or skeletal anchorage devices (TADs) for mandibular distalization [9–14]. In class III borderline cases, clinicians are called to find the best compromise between functional and aesthetic outcomes, with the latter which often turns in avoiding worsening of profile characteristics, which makes the treatment of these patients quite challenging.

Lower tooth extraction is sometimes indicated in moderate class III skeletal cases and may implicate first premolar or incisor extractions. The choice is influenced by many factors, such as the severity of the mandibular anterior crowding, the Bolton discrepancy, and negative overjet and overbite intensity [15].

This case report presents a skeletal class III orthodontic camouflage in an adult patient, illustrating the diagnostic process and the therapeutic options evaluated before opting for the lower first premolar extraction.

## 2. Case Report

### 2.1. Diagnosis and Etiology

**Figure 1:**
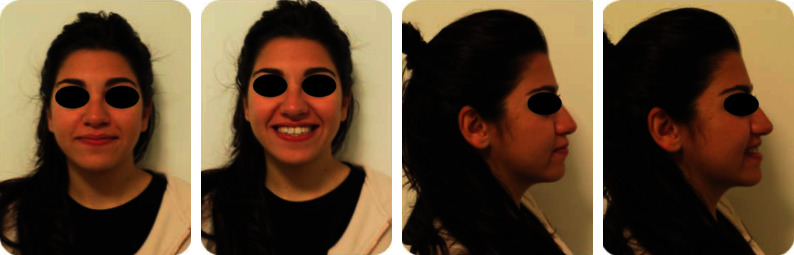
Pretreatment extraoral photographs.

**Figure 2:**
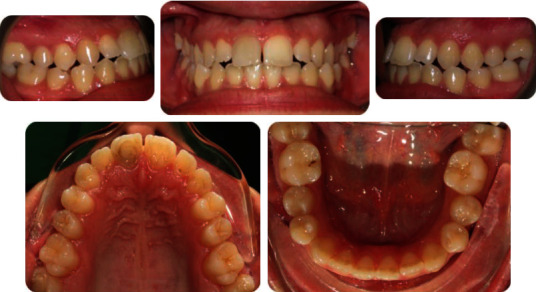
Pretreatment intraoral photographs.

**Figure 3:**
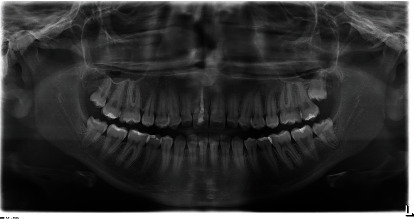
Pretreatment orthopantomogram.

**Figure 4:**
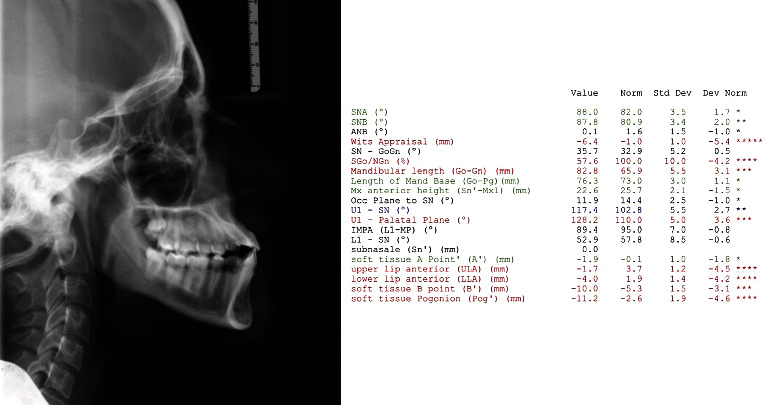
Pretreatment cephalometric analysis.

A 19-year-old female has come to orthodontic consultation with a chief complaint about the relapse of her occlusion after previous orthodontic treatment and the aesthetics of her smile. The patient had a moderate skeletal class III malocclusion with a flat profile, normal facial vertical dimension, and an adequate soft tissue projection with a slight lip strain (Figure 1). In the frontal view, the face had a good symmetry. The intraoral examination and the orthopantomogram showed spacing in the maxillary arch and severe crowding in the mandibular arch, with crossbite of 1.3 and 2.3 and edge-to-edge relationship of upper and lower premolars, with a discrepancy in upper and lower arch form (Figures 2 and 3). The spacing in the upper arch, with the crowding in the lower arch, could indicate an alteration of the Bolton index; however, this is not the case, as has been shown by the analysis of the plaster models. It seems more likely that this discrepancy is linked to the failure of the compensation carried out during the previous orthodontic treatment. The teleradiography and the consequent cephalometric analysis highlight the class III skeletal malocclusion in a subject tended to hyperdivergence, with an importance of upper incisor proclination and normal position of lower incisors (Figure 4). During anamnestic and clinical evaluation, no signs or symptoms of temporomandibular disorder (TMD) were described.

### 2.2. Treatment Objectives

The aims of the orthodontic treatment were the correction of maxillary spacing and mandibular crowding, normalization of upper incisors inclination, and coordination of the upper and lower arch form in order to correct the crossbite of teeth 1.3 and 2.3; moreover, improve the smile aesthetics without worsening the facial profile achieving those occlusal outcomes.

### 2.3. Treatment Alternatives

The first option was orthognathic surgery; however, the patient and her parents refused any surgical approach.

The second option was to treat the patient with four-premolar extraction (both upper and lower first or second premolars). This strategy would have permitted to correct both maxillary incisor proclination and mandibular crowding; however, this option would have probably worsened patients' facial aesthetics since the elimination of dental compensation would have accentuated the class III profile.

The third alternative was an orthodontic camouflage; this option could be achieved with class III elastics, with distalization of the lower arch through the use of TADs, or with mandibular first premolar extraction; however, the first two options for orthodontic camouflage were not suitable for this case: the first one because of the needs to correct the upper incisor proclination, which would surely be worsened by the use of class III elastics, and the second one because of the important and not very predictable lower distalization required which in any case would have required lower third molar extraction.

Consequently, the camouflage with first mandibular premolar extraction is the most reasonable option, capable of allowing the normalization of upper incisor inclination and the correction of mandibular crowding. This option would lead to a predictable improvement of facial aesthetics with a biological cost comparable to the lower arch distalization option.

Both patients' parents and the orthodontists preferred the latter option.

### 2.4. Treatment Progress

A 0.22 fixed appliance with preadjusted Roth prescription (American Orthodontics) has been bonded to all the erupted teeth on the buccal surface, except 3.4 and 4.4 that were extracted during treatment early stages. The same sequence of archwire was used for both arches: 0.014 heat-activated NiTi; 0.014 × 0.025 heat-activated NiTi; 0.019 × 0.025 heat-activated NiTi; and 0.019 × 0.025 SS. By the means of this archwire sequence alignment, leveling and the coordination of the maxillary and mandibular arch were achieved.

After 20 months of orthodontic therapy, the appliance was removed, lingual-bonded fixed retainers were applied to both maxillary and mandibular front teeth, and removable retainers were provided to the patient for the upper and lower arch [16].

### 2.5. Results

**Figure 5:**
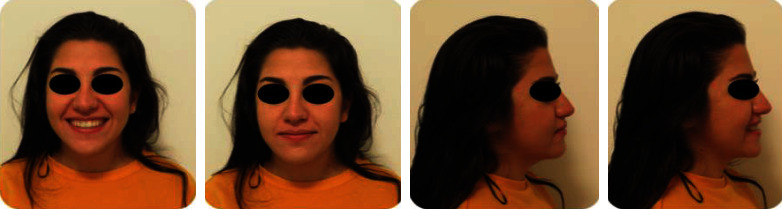
Posttreatment extraoral photographs.

**Figure 6:**
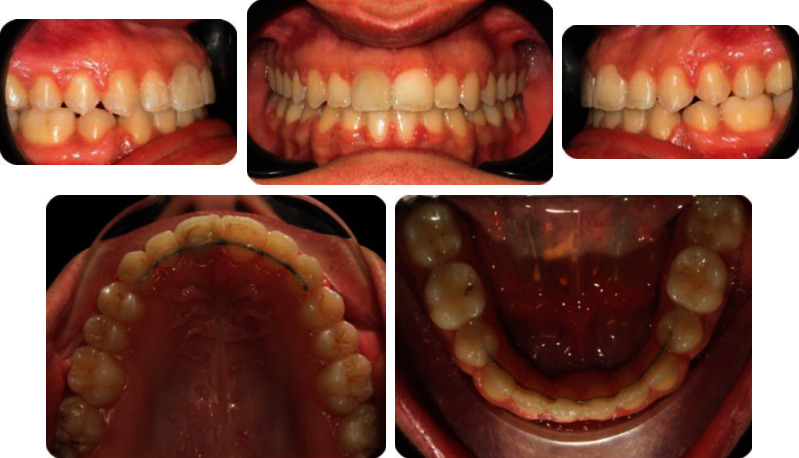
Posttreatment intraoral photographs.

**Figure 7:**
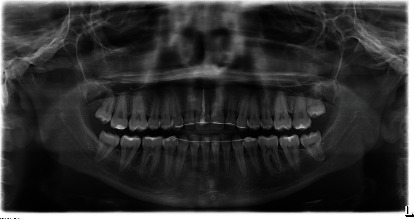
Posttreatment orthopantomogram.

**Figure 8:**
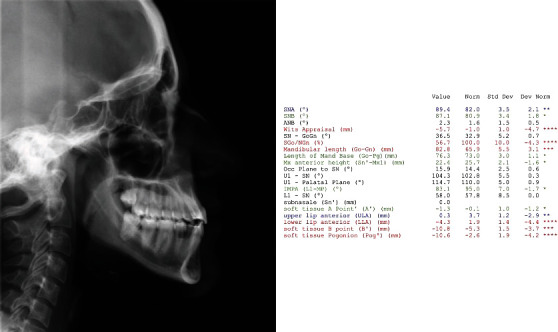
Posttreatment cephalometric analysis.

**Figure 9:**
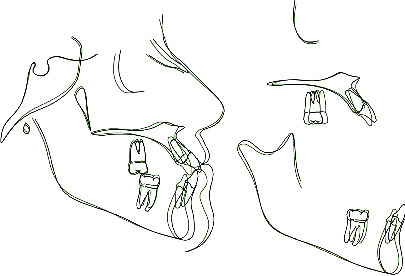
Pretreatment and posttreatment tracing superimposition.

The posttreatment photographs show the substantial improvement in the aesthetics of the patient (Figures 5 and 6). The posttreatment orthopantomogram displayed the good root parallelism achieved without root resorption (Figure 7). The posttreatment cephalometric analysis and the tracing superimposition illustrate the modifications obtained with the treatment (Figures 8 and 9). The posttreatment intraoral view shows the achievement of a functional canine class I canine relation with the normalization of overjet and overbite. The maxillary spacing correction has determined a normalization of upper incisor position; the lower crowding correction by means of extractions allowed correcting lower arch form and the crossbite of teeth 1.3 and 2.3. Furthermore, this extractive treatment option allowed compensating for the class III skeletal discrepancy improving the dental support of both upper and lower lips. Labial competence was improved with a reduction of the lip strain. Looking at the posttreatment lateral cephalogram, the orthodontic camouflage improved the dental relationship with a normalization of upper incisor inclination without a relevant lingualization of lower incisors; the skeletal facial pattern of the patient experienced a slight improvement.

**Figure 10:**
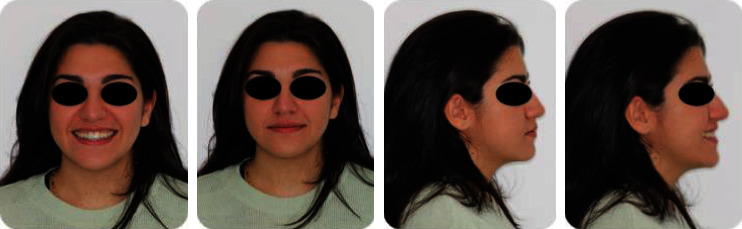
12-month follow-up, extraoral photographs.

**Figure 11:**
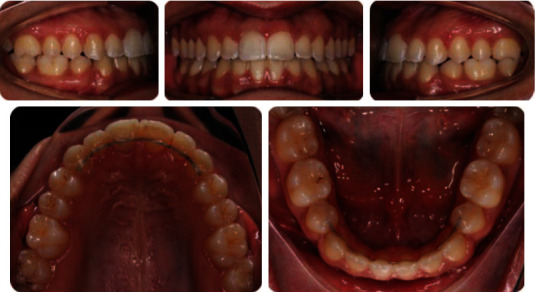
12-month follow-up, intraoral photographs.

Facial and intraoral view after 12 months of retention shows the stability of the results and the improvement of dental aesthetics thanks to the maturation of the tissues in the retention period. Tooth 1.1, which was dyschromic since the beginning of the treatment, due to a previous endodontic treatment, has been treated with the walking bleach technique (Figures 10 and 11) [17].

## 3. Discussion

Angle's Class III is notoriously one of the most complex malocclusions to understand, and it is also one of the most challenging malocclusions for which to develop an optimal treatment plan avoiding overtreatment or undertreatment [18–20].

The present case reports shows a borderline class III patient treated with an orthodontic camouflage achieved by lower first premolar extraction. As previously mentioned, this case left room for various therapeutic options, but the will to improve as much as possible the patient's aesthetics without resorting to orthognathic surgery led us to the decision to extract only lower first premolars. Since avoiding the extraction of upper first premolars would have allowed to achieve and maintain a good soft tissue balance, compensating for the lower lip protrusion; on the contrary, upper first premolar extraction and the consequent upper incisor retroclination would have worsened the upper lip projection, revealing the class III relationship. This aspect was crucial in the therapeutic choice, especially considering the patient's request not to excessively modify her facial aesthetics.

Furthermore, the extraction of lower first premolars, which were already in crossbite, allowed the clinician to correct the transversal discrepancy between upper and lower arches, achieving correct arch coordination.

In terms of treatment plan strategy, the class III malocclusion orthodontic camouflage by the means of the extraction of lower first premolars could be considered specular to upper premolar extraction in class II malocclusion orthodontic camouflage. The latter is a common therapeutic option in those situations in which class II relationship and increased overjet are accompanied by the need not to worsen the position of the lower teeth, as it could instead happen using class II mechanics; long-term evaluation of patients treated with orthodontic class II camouflage did not highlight any functional damage [21, 22]. Similarly, for class III orthodontic camouflage, there is no evidence that single arch extraction could lead to functional damage, not even with molar class III relationship if canine class I relationship is achieved.

What prompted us to share this clinical case is the need to review the concept of excellence in the orthodontic field, since in this case, notwithstanding the many valid therapeutic options and despite not having achieved all the standard canons of excellence [23], the biological sacrifice of two premolars made it possible to correct the malocclusion and significantly improve the patient's facial aesthetics. Therefore, orthodontic excellence should always be reviewed according to the initial clinical conditions, the needs and possibilities of the patients, and the skills of the clinician, aiming at tailor-made orthodontic excellence.

## 4. Conclusion

To date, there are no guidelines that can guide the orthodontist through the decision-making process for borderline orthodontic cases. The present case report shows how the choice of an effective treatment plan must necessarily take into account numerous clinical and extraclinical factors, aiming at orthodontic excellence, with a tailor-made treatment plan for each patient.

## Data Availability

The data used to support the findings of this study are available from the corresponding author upon request.
